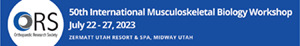# Abstracts from the 50th International Musculoskeletal Biology Workshop, July 22 – 27, 2023, Midway, Utah

**DOI:** 10.1002/jbm4.10827

**Published:** 2023-10-03

## Abstract